# PTEN Hamartoma Tumor Syndrome/Cowden Syndrome: Genomics, Oncogenesis, and Imaging Review for Associated Lesions and Malignancy

**DOI:** 10.3390/cancers13133120

**Published:** 2021-06-22

**Authors:** David D. Dragoo, Ahmed Taher, Vincenzo K. Wong, Ahmed Elsaiey, Nikita Consul, Hagar S. Mahmoud, Bilal Mujtaba, Nir Stanietzky, Khaled M. Elsayes

**Affiliations:** MD Anderson Cancer Center, Department of Diagnostic Imaging, University of Texas, Houston, TX 77030, USA; DDragoo@mdanderson.org (D.D.D.); artaher@mdanderson.org (A.T.); vkwong@mdanderson.org (V.K.W.); ahmed.elsaiey@gmail.com (A.E.); Nikita.Consul@bcm.edu (N.C.); hag.smahmoud@gmail.com (H.S.M.); bmujtaba@mdanderson.org (B.M.); NStanietzky@mdanderson.org (N.S.)

**Keywords:** Cowden syndrome, PTEN, multiple hamartoma syndrome, imaging review

## Abstract

**Simple Summary:**

In this manuscript, we present the associated imaging findings and imaging screening recommendations. Knowledge of the types of cancers commonly seen in Cowden syndrome and their imaging findings can aid in early tumor recognition during cancer screening to help ensure near-normal life spans in Cowden syndrome patients.

**Abstract:**

*PTEN* hamartoma tumor syndrome/Cowden syndrome (CS) is a rare autosomal dominant syndrome containing a germline *PTEN* mutation that leads to the development of multisystem hamartomas and oncogenesis. Benign tumors such as Lhermitte–Duclos disease and malignant tumors involving the breast, thyroid, kidneys, and uterus are seen in CS. Radiologists have an integral role in the comanagement of CS patients. We present the associated imaging findings and imaging screening recommendations. Knowledge of the types of cancers commonly seen in CS and their imaging findings can aid in early tumor recognition during cancer screening to help ensure near-normal life spans in CS patients.

## 1. Introduction

Cowden syndrome (CS) is rare, with an estimated prevalence of 1 in 200,000 individuals. It was first described by Lloyd and Dennis in 1963 and named for the family in which it was identified [1]. CS is a cancer predisposition syndrome inherited with germline mutations of the phosphatase and tensin homolog (*PTEN*) tumor suppressor gene [2]. This syndrome predisposes individuals to hamartomas that can involve any organ and to increased risk of malignancy [3]. CS is part of the PTEN hamartoma syndrome family, which includes a spectrum of other disorders with mutations in the *PTEN* gene [2].

We describe here the genomics and pathophysiology of CS, its common clinical presentations, and the radiologic–pathologic correlation of the most common associated benign and malignant tumors. We also discuss how radiologists and clinicians can approach the screening guidelines.

## 2. Genomics and Pathophysiology

**Figure 1:**
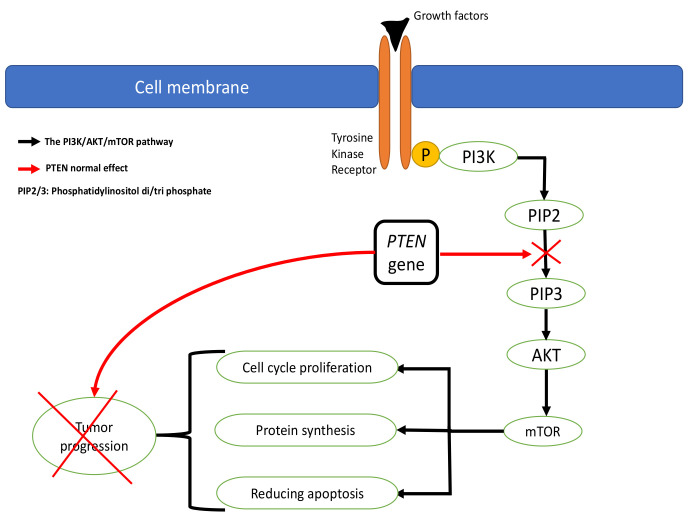
Illustration of the PI3K/AKT/mTOR pathway and regulation by the PTEN gene.

CS is usually inherited in an autosomal dominant fashion but arises from de novo *PTEN* mutations in up to 45% of cases. CS was recognized as a clinical entity in 1963 and found to be linked to *PTEN* mutations in 1997 [3]. Approximately 80% of CS patients have an identifiable mutation in the *PTEN* gene, which is located on chromosome 10 q23.3 [4]. The *PTEN* gene contributes to the control of apoptosis and the cell cycle by affecting the phosphatidylinositol 3-kinase (PI3K)/AKT/mammalian target of rapamycin (mTOR) pathways. The PI3K/AKT/mTOR pathway is an intracellular signaling pathway essential in regulating the cell cycle [3]. The pathway starts with phosphorylation of the PI3K enzymes to become phosphatidylinositol diphosphate (PIP2) and triphosphate (PIP3). The PIP3 stimulates AKT to activate the mTOR, which plays a primary role in protein synthesis, cell growth, proliferation, and reducing apoptosis (Figure 1). The *PTEN* gene affects the cell cycle by downregulating the PI3K/AKT/mTOR pathway, leading to decreased proliferation and cell survival, thus preventing tumor formation. Therefore, *PTEN* mutation is related to many cancers and tumor development. Patients who meet the clinical criteria for CS but do not demonstrate a mutation in the *PTEN* gene have been found to have germline mutations in *SDHB*, *SDHC*, and *SDHD*, resulting in “second hit” mutations that lead to heterozygous loss of *PTEN*. Hereditary pheochromocytoma and paraganglioma syndromes are also caused by these three genetic mutations. CS patients or those who have CS-like characteristics may also have associated hypermethylation of the KILLN promoter. Related conditions such as Bannayan–Riley–Ruvalcaba syndrome and segmental overgrowth, lipomatosis, arteriovenous malformation, and epidermal nevus (SOLAMEN) syndrome also share a mutation in the *PTEN* gene [3,5].

## 3. Clinical Presentation and Diagnostic Criteria

CS is typically a disease of young adults, presenting in the second to third decade of life; the average age of diagnosis is 39 years (range, 4–75 years) [6]. CS is slightly more common in females. The expressivity of CS is often variable, which makes diagnosis difficult. Mucocutaneous lesions such as oral papillomas, trichilemmomas, and acral keratoses are the most common presenting lesions in CS, occurring in 99% of patients by the third decade of life [2,6]. Typical cutaneous lesions consist of clusters of tiny papillomatous papules near the eyelids, forehead, nose, mouth, hands, or feet. Trichilemmomas may present as flesh-colored hamartomas at the hairline. Nontypical benign cutaneous lesions such as angiomas, dermal fibromas, lipomas, and neurofibromas may also occur. Malignant skin lesions such as melanoma, basal cell carcinoma, squamous cell carcinoma, and Merkel cell carcinoma are less likely [6].

Given that these mucocutaneous lesions are also prevalent in the general population, the International Cowden Consortium Criteria were developed in 1996 for the diagnosis of CS. Major criteria are defined as Lhermitte–Duclos disease (LDD), thyroid carcinoma, macrocephaly, and breast cancer (Table 1). Minor criteria include genitourinary tumors or malformations, lipomas, fibromas, mental retardation, fibrocystic disease of the breast, gastrointestinal hamartomas, and other thyroid lesions, such as goiter. Mucocutaneous lesions or palmoplantar keratosis can meet the criteria for the diagnosis of CS alone if six or more lesions are present (Table 1). An individual meets the criteria for a CS diagnosis if at least one of the following is present: adult LDD disease or a requisite number of mucocutaneous features, macrocephaly plus one other major criterion, one major and three minor criteria, or four minor criteria [4].

Multiple updates to the 1996 consensus criteria have been proposed over the years after the discovery that the *PTEN* gene is associated with CS. The recommended updates include adding endometrial cancer to the major criteria and renal cell carcinoma (RCC) to the minor criteria [7]. In 2013, Pilarski et al. conducted a review of the literature to update the clinical features associated with *PTEN* mutations and the associated clinical syndromes. The revised criteria established both major and minor criteria for the operational diagnosis of PTEN hamartoma syndromes in both individuals and families (Table 2). An operational diagnosis in an individual is made in the presence of three or more major criteria, one of which must be macrocephaly, LDD, or gastrointestinal hamartomas, or in the presence of a combination of two major and three minor criteria. An operational diagnosis within a family is made when one family member (1) meets the revised diagnostic criteria or (2) has a *PTEN* mutation and one of the following: two major criteria with or without minor criteria, one major and two minor criteria, or three minor criteria [7]. The National Comprehensive Cancer Network (NCCN) has subsequently adopted the revised PTEN hamartoma syndrome clinical criteria as the basis for its version 2.2021 CS/PTEN hamartoma syndrome management guidelines [8,9].

Criteria for determining the need for genetic testing for the *PTEN* mutation have also been developed by the NCCN. These criteria are based on clinical diagnostic criteria. Once a clinical and genetic diagnosis of *PTEN* hamartoma syndrome has been made, NCCN management and screening guidelines should be followed [8,9].

## 4. Associated Benign Pathologies

**Figure 2:**
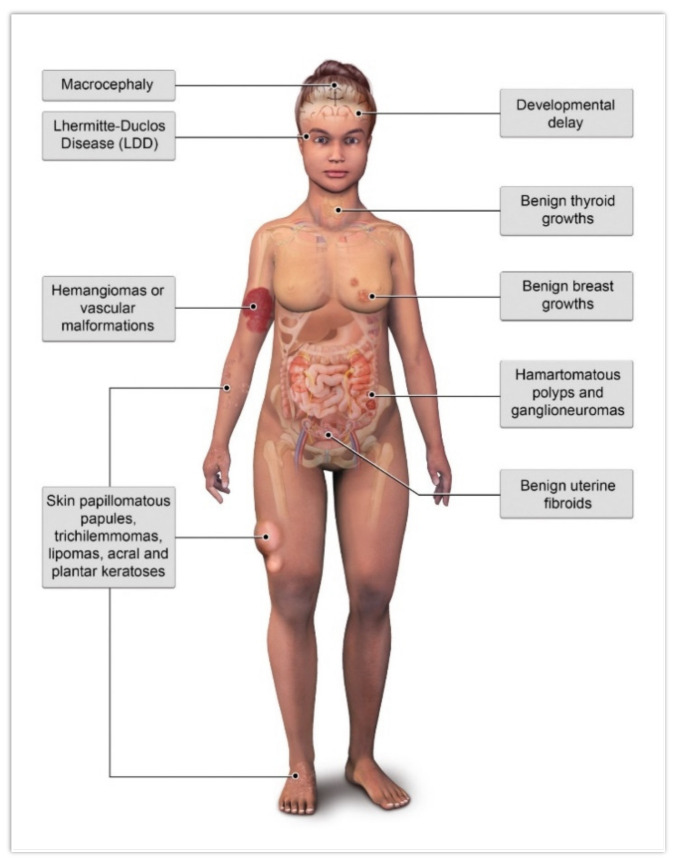
Illustration of common benign pathologies associated with Cowden syndrome.

There are several benign pathologies associated with CS, including macrocephaly, LDD, mucocutaneous lesions, developmental delay, benign thyroid growths, benign breast growths, hamartomatous polyps of the gastrointestinal tract, uterine fibroids, and hemangiomas/vascular malformations (Figure 2). Of these benign findings, only LDD and macrocephaly are considered major findings [4].

## 5. Lhermitte–Duclos Disease (LDD)

**Figure 3:**
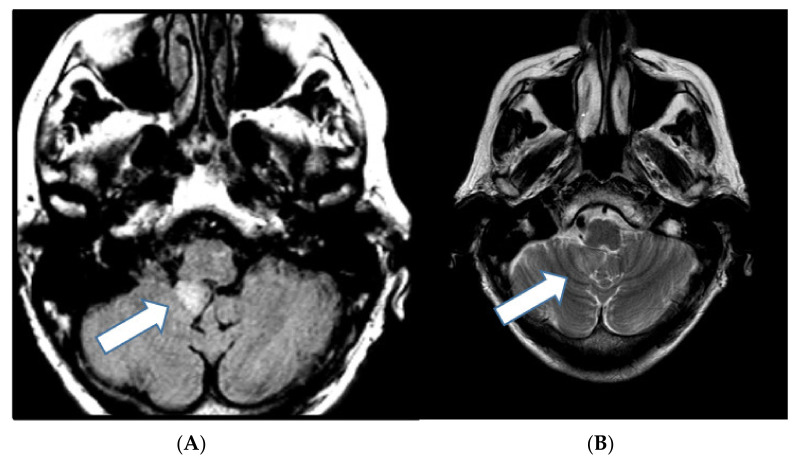
A 66-year-old woman with a history of clinical Cowden syndrome and pathologic *PTEN*-mutation-associated meningioma presenting with headache. (**A**) Axial FLAIR magnetic resonance image demonstrates a hyperintense oval mass measuring 13 mm at the inferior right cerebellum (arrow), encroaching on the fourth ventricle. (**B**) T2-weighted images demonstrate classic “tigroid” appearance representing dysplastic gangliocytoma (Lhermitte–Duclos disease).

LDD is a rare pathologic, nonmalignant condition of the central nervous system consisting of a dysplastic gangliocytoma of the cerebellum. LDD is a major criterion for the diagnosis of CS. LDD is typically diagnosed in the third to fourth decade of life and has a *PTEN* mutation, distinguishing it from the pediatric form, which lacks this mutation. Clinically, it presents with headaches, cerebellar ataxia, and visual problems. This lesion can lead to increased intracranial pressure, which may be life threatening [2]. Magnetic resonance imaging (MRI) of the brain, with or without contrast, is the preferred diagnostic imaging modality. LDD manifests as a unilateral cerebellar lesion with T2/FLAIR hyperintensity and widening of the cerebellar folia, which appears as parallel striations, causing the characteristic “tigroid” appearance (Figure 3). Enhancement is rare [10]. Pathologically, it is characterized by the presence of dysplastic ganglion cells, which disrupt the normal cerebellar cellular organization. Treatment is surgical resection [2].

## 6. Vascular Malformations

**Figure 4:**
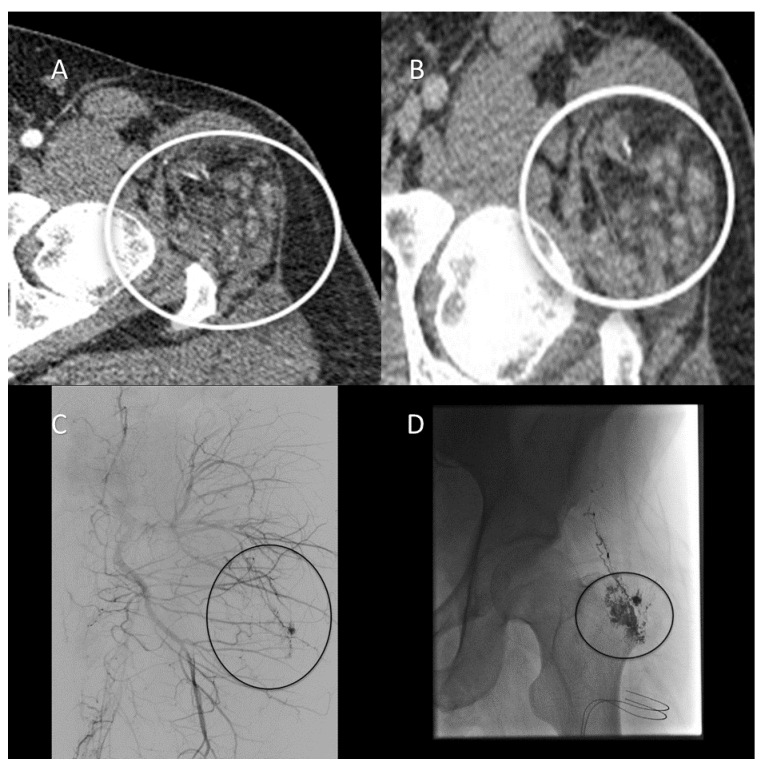
A 37-year-old man with Cowden syndrome and follicular thyroid cancer with a history of left hip pain and palpable abnormality. (**A**) Arterial-phase computed tomographic (CT) angiography of the lower extremities demonstrates an arterially enhancing lesion containing abnormal vasculature involving the left inferior gluteus medius muscle adjacent to the left hip supplied by branches of the lateral circumflex femoral artery (circle). (**B**) Venous-phase CT angiography demonstrates persistent enhancement consistent with an arteriovenous malformation (AVM). (**C**) Pre-embolization digital subtraction angiogram demonstrates an AVM supplied by branches of the left superior gluteal and lateral circumflex femoral arteries (circle). (**D**) Postglue embolization angiogram demonstrates stasis within AVM (circle).

Nonspecific vascular malformations including arteriovenous malformations and hemangiomas have been reported in CS patients more commonly than in the general population. Rates of vascular malformations in the CS population range from 18% to 34%, versus 5% to 10% in the general population [4]. Vascular anomalies are considered to be a minor diagnostic criterion within the NCCN revised clinical criteria for the diagnosis of PTEN hamartoma syndrome [8]. It has been reported that the potential mechanism for vascular lesions in this population is related to PTEN’s role in controlling angiogenesis associated with vascular endothelial growth factor. Clinically, these vascular lesions tend to present as multifocal, intramuscular, high-flow lesions or intracranial developmental abnormalities [4]. Radiographically, vascular malformations can be evaluated with computed tomographic (CT) angiography, Doppler ultrasonography, MRI angiography, or catheter-based angiography. Across modalities (CT, MRI, and angiography), vascular malformations appear as heterogenous lesions with abnormal vasculature that often have adjacent soft tissue abnormalities with possible phleboliths and varying degrees of postcontrast enhancement, depending on the rate of flow within the lesion [11,12] (Figure 4).

## 7. Associated Malignant Pathologies

**Figure 5:**
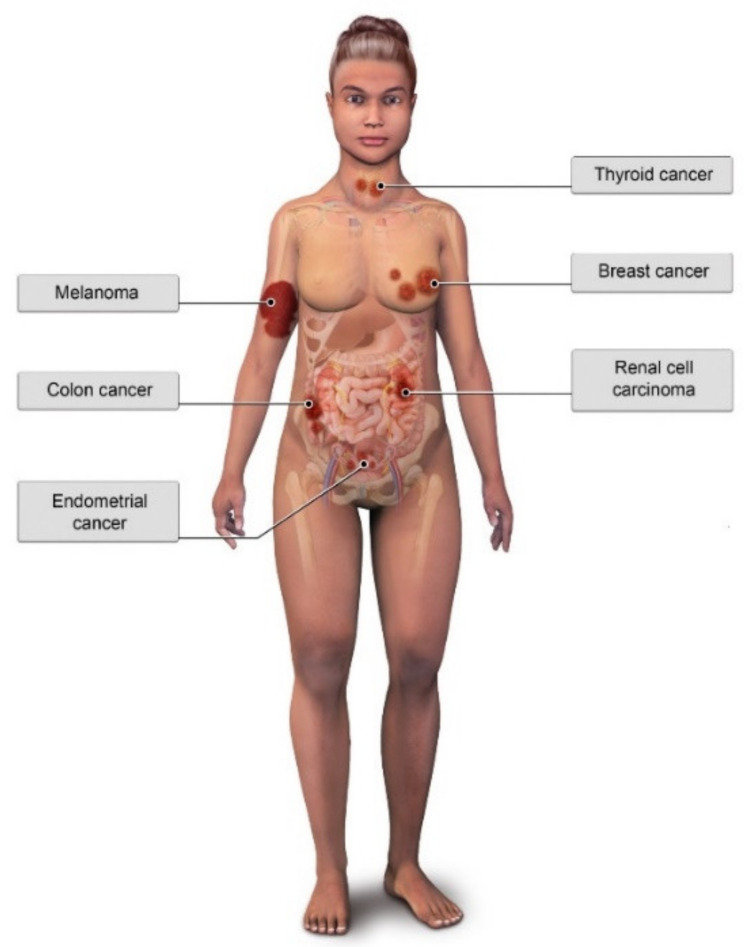
Illustration of common malignant pathologies associated with Cowden Syndrome.

Due to germline *PTEN* mutations, CS patients have a significantly elevated lifetime risk of cancer—specifically breast, thyroid, endometrial, and renal malignancies—when compared with the general population. CS patients also have a slightly elevated risk of colorectal cancers and melanoma [13] (Figure 5).

### 7.1. Breast Cancer

**Figure 6:**
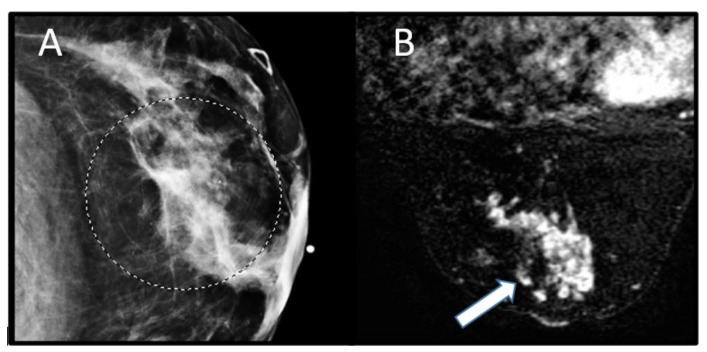
A 37-year-old woman with a clinical diagnosis of Cowden syndrome (negative for *PTEN* mutation) and history of thyroid cancer at age of 24 and Wilms tumor at age 5, presenting with a palpable left breast mass. (**A**) Mammographic examination demonstrates an irregular mass in the 3 o’clock position centered 1 cm from the nipple with microcalcifications (circle). (**B**) Postcontrast, fat-saturated magnetic resonance image of the breast shows mass enhancement involving 3 of 4 quadrants due to multiple confluent masses (arrow), which was pathologically found to represent invasive mammary carcinoma with predominantly lobular differentiation and focal ductal component.

Breast cancer is the most common component cancer of CS. It is reported that women with CS have a 25% to 50% risk of breast cancer development, compared with 12% of women in the general population. Breast cancer in males affected with CS is exceedingly rare, with only two cases reported in the literature. The average age of diagnosis in patients with CS ranges from 38 to 46 years old [4]. Although difficult to estimate, the lifetime risk of breast cancer in women with CS is approximately 85%, which is similar to that of BRCA carriers [13]. There are no distinguishing differences in the imaging or histopathologic appearance of breast cancer in CS patients compared with the general population [4]. On MRI, breast cancers typically demonstrate mass or nonmass enhancement on T1-weighted images, with noncircumscribed or irregular margins and suspicious washout or plateau enhancement kinetics [14] (Figure 6).

### 7.2. Thyroid Carcinoma

**Figure 7:**
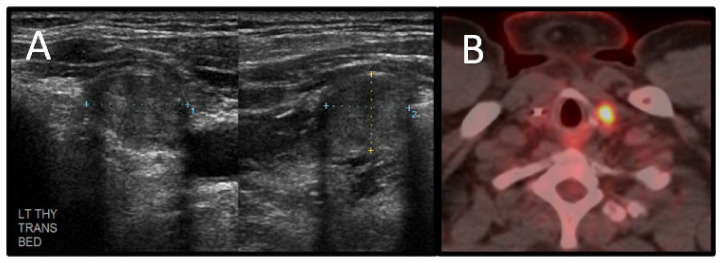
A 43-year-old man with a history of Cowden syndrome and follicular thyroid cancer diagnosed at age 30. The patient underwent thyroidectomy at diagnosis. He subsequently was diagnosed with primary lung cancer and was found to have a nodule in the resection bed on routine surveillance. (**A**) Grayscale ultrasound demonstrates a well-circumscribed nodule involving the left thyroid resection bed suggestive of recurrence. (**B**) Nodule shows increased FDG uptake on positron emission tomography/computed tomography. The nodule was biopsied and found to represent follicular thyroid carcinoma.

Thyroid carcinoma is the second most common component cancer of CS. Thyroid cancers of epithelial origin are found in one-third of patients with clinical CS or *PTEN* mutations. The risk of developing thyroid carcinoma among patients with CS is reportedly 3–10%, whereas the risk among the general population is less than 1%. The estimated lifetime risk of thyroid cancer in CS is 38%. The mean age of diagnosis for thyroid carcinoma among CS patients is 32 years old [3,7,15]. Histopathologically, thyroid carcinomas in CS are exclusively follicular or papillary, with the majority being follicular. Radiologically, the diagnosis of thyroid carcinoma can be difficult in patients with CS because of the other benign manifestations of thyroid disease that occur in CS, including multinodular goiter, solitary nodules, and Hashimoto’s thyroiditis. These benign processes are present in 50–70% of CS patients [3,15]. In general, nodules highly suspicious for thyroid cancer tend to appear on ultrasonography as solid hypoechoic nodules with irregular margins that lack a hypoechoic halo surrounding the nodule and internal vascularity with possible microcalcifications [16]. There are no known specific imaging features that differentiate papillary and follicular thyroid cancers in CS from those in the general population (Figure 7).

### 7.3. Endometrial Cancer

**Figure 8:**
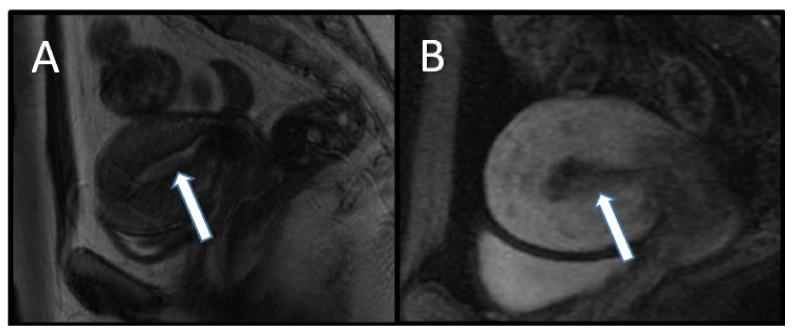
An 18-year-old woman with Cowden syndrome who underwent surveillance biopsy for dysmenorrhea and irregular menstrual bleeding. Biopsy-proven grade 1 endometrioid endometrial carcinoma was found, and staging magnetic resonance imaging (MRI) was performed. (**A**) Sagittal T2-weighted and (**B**) contrast-enhanced T1-weighted MR images show endometrial thickening (arrows) measuring approximately 2 cm. The patient subsequently underwent hysterectomy, and the pathologic diagnosis was upgraded to grade 2 endometrioid endometrial cancer.

The lifetime risk for development of endometrial cancer is 5–10% in women with CS. The risk of endometrial cancer in the general population is approximately 2.6% over a lifetime [4]. The relative risk of endometrial cancer dramatically increases in women with CS who are 25 to 50 years old. There are no distinguishing histopathological characteristics of endometrial cancer in CS compared with the general population [3]. On transvaginal ultrasonography, endometrial carcinoma appears as thickening of the endometrium that has a heterogeneous echogenicity with potentially irregular margins and internal vascularity. The MRI appearance of endometrial carcinoma is that of a thickened endometrium that is T1 isointense, T2 hyperintense, or heterogeneous relative to normal endometrium, and enhances earlier than normal endometrium and later than adjacent myometrium on postcontrast imaging. Additionally, endometrial cancer demonstrates restricted diffusion (high signal) on diffusion-weighted images and a low signal on the apparent diffusion coefficient map [17] (Figure 8).

### 7.4. Renal Cell Carcinoma

**Figure 9:**
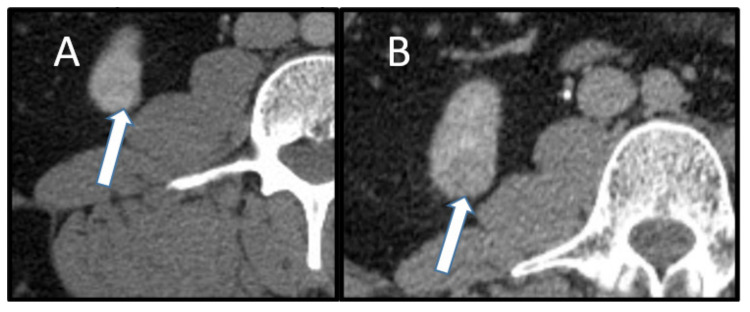
A 39-year-old man with a history of Cowden syndrome, follicular thyroid cancer, and lung cancer. Axial contrast-enhanced computed tomography demonstrates (**A**) corticomedullary phase with subtly enhancing 1.9 cm lesion (arrow) in the inferior right kidney, and (**B**) delayed imaging shows washout (arrow). This was pathologically proven to represent clear cell renal carcinoma. An additional 1.6 cm biopsy-proven clear cell renal cell carcinoma with the same imaging features was present in the superior pole left kidney.

RCC is one of the minor criteria established for the diagnosis of CS. Until recently, the risk of RCC in CS was unknown; however, recent research places the lifetime risk as high as 34% in the setting of *PTEN* mutation. The age of onset of RCC in the CS population is around 40 years old [7]. Histologic review of RCC in CS shows the majority of these cancers to be papillary or chromophobe neoplasms; however, clear cell neoplasms can occur (Figure 9). On CT and MRI, papillary RCC tends to be hypoenhancing compared with the adjacent normal renal parenchyma [18]. Chromophobe RCC often demonstrates homogenous enhancement on CT or MRI and may sometimes have a “spoke wheel pattern” that is more commonly associated with oncocytomas [19].

## 8. Imaging Screening Guidelines for Cowden Syndrome

According to the NCCN guidelines (v2.2021), imaging surveillance is recommended to screen CS patients for breast, thyroid, and renal cancers. All imaging recommendations are class 2A evidence. Currently, the NCCN does not recommend transvaginal ultrasonography (TVUS) as a screening modality for endometrial carcinoma. In premenopausal women, TVUS is not recommended because of the cyclical changes in endometrial stripe thickness. In postmenopausal women, TVUS can be considered at the clinician’s discretion; however, it is neither sensitive nor specific enough to elicit a supportive recommendation. Breast cancer screening for CS-affected women should take the form of annual mammography and screening breast MRI with contrast beginning at age 30–35 years or 5–10 years prior to the earliest known case of breast cancer within the family. Thyroid cancer screening ultrasonography in CS patients is recommended to begin at the age of 7 and continue annually. Screening ultrasonography for RCC should begin at the age of 40 and be repeated every 1–2 years [8] (Table 3).

## 9. Conclusions

Given the wide spectrum of benign and malignant tumors that may develop over the lifetime of a patient with CS, screening guidelines established by the NCCN place radiologists at the forefront of CS patient management. An understanding of the types of cancers commonly seen in CS and their imaging findings can aid in early tumor recognition to help ensure near-normal life spans in CS patients.

## Figures and Tables

**Table 1:** Major and minor diagnostic criteria established by the International Cowden Syndrome Consortium for the diagnosis of Cowden Syndrome.

Major Criteria *	Minor Criteria
Lhermitte–Duclos disease *	Genitourinary tumors (RCC) or malformations
Thyroid carcinoma	Lipomas
Macrocephaly *	Fibromas
Breast cancer	Mental retardation
	Fibrocystic disease of breast
	Gastrointestinal hamartomas
	Other thyroid lesions, such as goiter
	Mucocutaneous lesions or palmoplantar keratosis can meet the criteria for CS alone if six or more lesions are present

* Patients need to have two major criteria to be diagnosed with Cowden disease, and one of the two must be either Lhermitte–Duclos disease or macrocephaly.

**Table 2:** Revised clinical diagnostic criteria for PTEN hamartoma syndrome adopted by NCCN, version 2.2021.

Revised Clinical Diagnostic Criteria for PTEN Hamartoma Syndrome *	
Major Criteria	Minor Criteria
Breast cancer	Autism disorder
Endometrial cancer (epithelial)	Colon cancer
Thyroid cancer	Esophageal glycogenic acanthoses
Gastrointestinal hamartomas (includes ganglioneuromas but excludes hyperplastic polyps, ≥3)	Lipomas
Lhermitte–Duclos disease (adult)	Intellectual disability (IQ ≤ 75)
Macrocephaly (≥97th percentile)	Renal cell carcinoma
Macular pigmentation of the glans penis	Testicular lipomatosis
Multiple mucocutaneous lesions (any of the following): -- Multiple trichilemmomas (≥3, at least one biopsy proven) -- Acral keratosis (≥3 palmoplantar keratotic pits and/or acral hyperkeratotic papules) -- Mucocutaneous neuromas (≥3) -- Oral papillomas (particularly on tongue and gingiva), multiple (≥3), or biopsy proven or dermatologist diagnosed	Thyroid cancer (papillary or follicular)
	Thyroid structural lesions (e.g., adenoma, multinodular goiter)
	Vascular anomalies/malformations (including multiple developmental venous anomalies)

* Pilarski R, Burt R, Kohlman W, et al. Cowden syndrome and the PTEN hamartoma syndrome: systematic review and revised diagnostic criteria. J Natl Cancer Inst 2013; 105: 1607–1616.

**Table 3:** National Comprehensive Cancer Network (NCCN), v2.2021, cancer screening recommendations for patients with Cowden syndrome.

Associated Cancer	Surveillance/Risk Reduction Method	Interval	Starting Age, Years	Evidence Category
Breast cancer	MRI	Yearly ^a,f^	30–35 ^e^	2A
	Mammography	Yearly ^a,f^	30–35 ^e^	2A
	Risk-reducing surgery offered	–	–	2A ^e^
Thyroid cancer	Ultrasonography	Yearly	7	2A
Renal cancer	Ultrasonography	Every 1–2 years	40	2A
Colorectal cancer	Colonoscopy	Every 5 years ^b^	35 ^d^	2A
Melanoma	Skin examination	Yearly	30	2A
Endometrial cancer	Imaging surveillance not recommended ^c^	–	–	–

^a^ The appropriateness of imaging modality and scheduling interval is still under study. ^b^ If the patient is symptomatic or polyps are found, more frequent surveillance should occur at the discretion of the gastroenterologist. ^c^ Transvaginal ultrasound can be considered in postmenopausal women at the clinician’s discretion (not recommended for premenopausal women). ^d^ Begin surveillance 5–10 years prior to the earliest known onset of malignancy in any family member. ^e^ Discuss prophylactic mastectomy in patients with pathogenetic disease/variants. For clinical Cowden syndrome, consideration of risk-reducing surgery should be based on family history. ^f^ For patients >75 years old, management should be determined on an individual basis.

## Data Availability

Data available on request.
